# The effects of component‐specific treatment compliance in individually tailored internet‐based treatment

**DOI:** 10.1002/cpp.2351

**Published:** 2019-02-22

**Authors:** Martin Kraepelien, Kerstin Blom, Nils Lindefors, Robert Johansson, Viktor Kaldo

**Affiliations:** ^1^ Department of Clinical Neuroscience, Centre for Psychiatry Research Karolinska Institutet Stockholm Sweden; ^2^ Department of Psychology Stockholm University Stockholm Sweden; ^3^ Division of Psychiatry Haukeland University Hospital Bergen Norway; ^4^ Department of Psychology, Faculty of Health and Life Sciences Linnaeus University Växjö Sweden

**Keywords:** adherence, co‐morbidity, depression, internet‐based treatment, tailoring, treatment compliance

## Abstract

The objective of this study was to explore the effects of treatment compliance in a guided individually tailored internet‐based treatment (TAIL) in relation to depression and co‐morbid symptoms. Compliance with the homework in the different treatment components in TAIL, each aimed at a specific condition, was rated for 207 participants by independent assessors. Six subgroups (*n* = 34–131) were constructed consisting of participants with co‐occurring symptoms of worry, panic, social anxiety, stress, insomnia, or pain. For each group, hierarchical regression was used to investigate whether the total sum of compliance points, Overall Compliance, predicted reductions in depression and in condition‐specific symptoms. Also, in each subgroup, it was tested whether working with specific treatment components, Specific Compliance, predicted reduction of the targeted symptoms. Overall Compliance predicted 15% of the reduction in depression symptoms. For participants with worry, panic, social anxiety, stress, or insomnia, Overall Compliance also predicted symptom reductions in that specific condition. Specific Compliance predicted reduction in the targeted symptoms for participants with social anxiety, stress, and insomnia. Specific Compliance with stress and insomnia components also predicted reductions in depression. Our results strengthen the importance of compliance in internet‐based treatments. Because compliance with stress and insomnia components was particularly important for broad symptom reductions, these conditions should not be ignored when treating patients with co‐morbid symptoms.

**Abbreviations**
TAIL: guided individually tailored internet‐based treatment for depression
MADRS‐S: Montgomery–Åsberg Depression Rating Scale – Self‐rated
PSWQ: Penn State Worry Questionnaire
PDSS‐SR: Panic Disorder Severity Scale – Self report
LSAS‐SR: Liebowitz Social Anxiety Scale – Self‐Rated
PSS‐10: Perceived Stress Scale – 10 item
ISI: Insomnia Severity Index
MPI: Multidimensional Pain Inventory

**Key Practitioner Message**

- Treatment compliance was rated in an individually tailored internet treatment.
- Overall Compliance predicted symptom reductions in all conditions, except pain.
- Specific Compliance predicted reductions in social anxiety, stress, and insomnia.
- Compliance with stress and insomnia components predicted reductions in depression.

## 1 INTRODUCTION

### 1.1 Treatment compliance as a predictor of outcome

In traditional face‐to‐face psychological treatment, there is a wealth of research on treatment compliance, and there is evidence of a correlation between compliance with homework assignments and symptom reductions (Glenn et al., 2013; Mausbach, Moore, Roesch, Cardenas, & Patterson, 2010). In a study of the relations between compliance and the effects of a cognitive behavioural therapy for zdepression, the authors concluded that homework compliance led to reductions in depression symptoms (Burns & Spangler, 2000). The design in this study was, however, correlational and could not rule out other causal mechanisms (Kazantzis, Ronan, & Deane, 2001). When measuring treatment compliance in face‐to‐face treatment, it has been suggested that it is important to include both quantity and quality of performed homework in contrast to just treatment quantity (Kazantzis, Brownfield, Mosely, Usatoff, & Flighty, 2017; Lebeau, Davies, Culver, & Craske, 2013). A recent meta‐analysis showed that the quality of homework in face‐to‐face treatment was a strong predictor of symptom reductions (Kazantzis et al., 2016). There are a few studies investigating whether compliance with specific components or techniques in an intervention, hypothesized to have a specific effect on symptoms, are more important than compliance with other components or techniques. For example, compliance with the exposure component seems to be more important than psychoeducation and self‐monitoring in order to decrease panic and anxiety in a panic disorder treatment (Cammin‐Nowak et al., 2013). Furthermore, in a guided self‐help insomnia treatment, compliance with the specific techniques of sleep restriction and stimulus control mediated the large extra effect that therapist guidance had in in contrast to unguided self‐help (Kaldo, Ramnerö, & Jernelöv, 2015). This suggests that the effects of compliance with specific treatment components can be very important and should be further explored.

In the field of internet interventions, there is a lack of compliance research (including both quantity and quality), as a predictor of improvement. One well‐established predictor of effect in internet‐based interventions is quantitative adherence to the treatment programme. Quantitative adherence has been defined both as how much of the intervention material the individuals were exposed to (Christensen, Griffiths, & Farrer, 2009; Fuhr et al., 2018) and similarly as how much time the participants spent with the intervention (Sieverink, Kelders, & van Gemert‐Pijnen, 2017). However, by defining adherence as equal to quantitative exposure to the intervention material, the aspect of actually using the components in the intervention as intended, in a clinically meaningful way, is missing.

### 1.2 Internet‐based psychological treatments

There is a growing interest in offering internet‐based interventions as a way of improving access to psychological treatments in the primary care population, for example, in the United Kingdom (Richards et al., 2018). Therapist‐guided internet‐based treatments result in large and clinically relevant improvements on depression in clinical trials (Karyotaki et al., 2018) and other conditions (Andersson, Cuijpers, Carlbring, Riper, & Hedman, 2014) and have shown effectiveness for several conditions when implemented in routine care (Andersson & Hedman, 2013; Hedman et al., 2014; Nordgreen, Gjestad, Andersson, Carlbring, & Havik, 2018; Titov et al., 2017).

### 1.3 Internet‐based treatments addressing co‐morbid conditions

Because of the high rates of co‐morbid conditions in patients with depression (Sundquist, Ohlsson, Sundquist, & Kendler, 2017), internet‐based treatments have been developed that address depression, anxiety, and stress simultaneously (Titov et al., 2015). A related strategy is individually tailored internet‐based treatments, where a mandatory generic treatment component is followed by an individual treatment plan, consisting of a mix of specific components taken from evidence‐based disorder‐specific treatments (Johansson et al., 2012). In the study by Johansson and colleagues, the individually tailored internet‐based treatment was effective for depressive symptoms, but specific effects on co‐morbid conditions were not examined.

### 1.4 The REGASSA study

The current study is based on data from the REGASSA study. The REGASSA study was a randomized controlled trial with three arms, aimed at participants with at least mild depressive symptoms, in primary care. An individually tailored internet‐based treatment for depression and co‐morbid conditions with brief online therapist guidance (TAIL) was compared with physical exercise and with treatment as usual in primary care. For a description of the TAIL treatment, please see Table 1 and a description in the supplementary material of a previous paper about the REGASSA study (Kraepelien et al., 2018). TAIL was more effective than treatment as usual in reducing symptoms of depression (Hallgren et al., 2015) as well as in improving psychological functioning and sleep (Strid, Andersson, Forsell, Öjehagen, & Lundh, 2016). TAIL is likely a cost‐effective treatment alternative from a health care perspective (Kraepelien et al., 2018) but did not reduce the participants' sick‐leave status compared with treatment as usual (Kaldo et al., 2018). The effects of TAIL on depression was found noninferior to disorder‐specific internet‐based treatment for depression in a benchmarking study (Kraepelien, Forsell, et al., 2018). However, noninferiority compared with disorder‐specific treatment could, in the benchmarking analysis, not be established for social anxiety or panic disorder. This could partly be because participants differed in their compliance with different treatment components in TAIL.

**Table 1:** Modules available for the different specific conditions

Mandatory transdiagnostic modules	Optional worry modules	Optional panic modules	Optional social anxiety modules	Optional stress modules	Optional insomnia modules	Optional pain modules	Optional depression‐ and non‐specific modules
•**Intro 1** Values and goals •**Intro 2** Behavioural activation •**Intro 3** Avoidance behaviours and exposure •**Last module** Relapse prevention and planning for the future	•**Worry 1** Functional analysis of worry •**Worry 2** Worry time •**Worry 3** Worry exposure •**Applied relaxation** Relaxation training •**Problem solving** Structured problem solving	•**Panic 1** Panic attacks and breathing •**Panic 2** Interoceptive exposure •**Panic 3** Agoraphobic exposure	•**Social anxiety 1** Social anxiety exposure •**Social anxiety 2** Self‐focus and safety behaviours	•**Stress** Planned recovery •**Perfectionism** Imperfectionism exposure •**Assertiveness** Say no to tasks •**Handle job problems** Time management at work •**Focus shifting** Focus shifting training •**Applied relaxation** Relaxation training •**Mindfulness** Mindfulness training	•**Sleep problems 1** Sleep diary, winding down, challenge thoughts about sleep •**Sleep problems 2** Sleep restriction and stimulus control	•**Pain 1** Pain psychoeducation and recognize risk situations •**Pain 2** Plan activity despite pain •**Applied relaxation**	•**Negative automatic thoughts** Challenge thoughts •**Depression 1** Behavioural activation •**Depression 2** Challenge negative automatic thoughts •**Healthy habits** Schedule new habits •**Exercise** Schedule exercise •**Contact with authorities** Schedule contacts •**Returning to work** Plan the return to work •**Looking for a new job** Schedule job searching

*Note*. Applied relaxation was used in the optional modules for worry, stress, and pain. As a result, rated compliance with the applied relaxation module was included in the specific, and excluded from the non‐specific, compliance scores for these three conditions.

Because TAIL includes a wide range of different components targeting specific conditions and symptoms and is used for a co‐morbid patient group, it constitutes a very good base for exploring the effects that both component‐specific compliance and overall compliance have on treatment outcome.

### 1.5 Aim

The aim of this study was to explore if compliance with all homework components in the TAIL treatment (Overall Compliance) and compliance with the condition‐specific homework components (Specific Compliance) predict reductions in condition‐specific symptoms and in symptoms of depression, in patients with depression and co‐occurring conditions who participated in the internet treatment arm of the REGASSA study.

## 2 METHOD

The REGASSA study was approved by the Regional Ethics Review Board in Stockholm, Sweden (2010/1779‐31/4).

### 2.1 Participants

**Figure 1:**
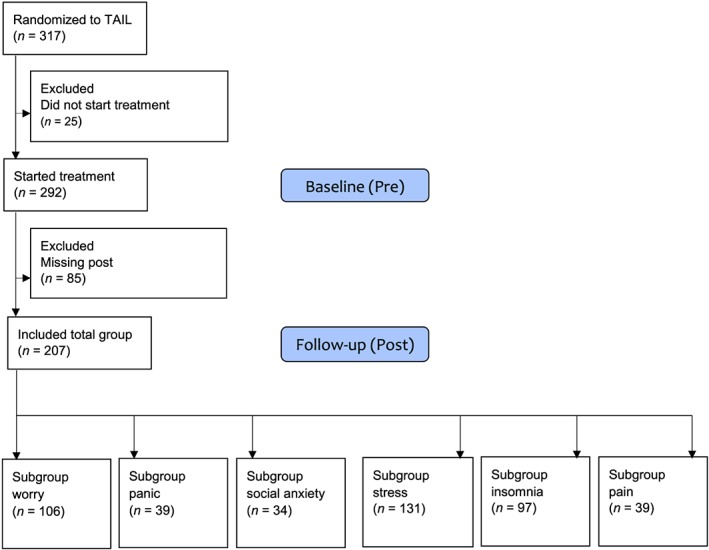
Flow chart of study participants and analysed subgroups. The subgroups of specific conditions may overlap, that is, a participant with both insomnia and pain is included in both subgroups [Colour figure can be viewed at wileyonlinelibrary.com]

Recruitment to the REGASSA study was done at primary care level in six county councils in Sweden between February 2011 and December 2012. Adults over 18 years who had depressive symptoms, defined as having at least 10 points on the Patient Health Questionnaire (Kroenke, Spitzer, & Williams, 2001), were included. Patients in need of specialist psychiatric treatment, having substance dependency or poor understanding of Swedish, were excluded. For more details on recruitment, see the earlier reported results from the main results of the REGASSA trial (Hallgren et al., 2015). One third (*n* = 317) of the participants in the trial were randomized to the internet treatment TAIL, and the 207 of these with post‐treatment self‐report measures were included in the current study. Please see Figure 1 for the study flow chart and Table S1 for characteristics of participants with missing postdata.

For the purpose of the analyses in this study, we constructed six subgroups representing the specific conditions targeted in the treatment and placed each participant in one or several subgroups. In order to decide which subgroups a participant belonged to we used (a) the participant's self‐report that they recognized disorder‐specific symptoms described in a brief text that was distributed during the beginning of the treatment, (b) the participant's rating of the severity of these symptoms, and (c) the participant's score on the symptom scales that they filled out pretreatment (see Section 2.2 for cut‐off scores)

### 2.2 Measures

The self‐report measures were collected via the internet pretreatment and post‐treatment, 12 weeks later. Primary outcome was depression symptoms, assessed with the Montgomery–Åsberg Depression Rating Scale – Self‐rated (MADRS‐S; Svanborg & Asberg, 1994). Secondary outcomes were symptoms of the six co‐occurring conditions: Worry was assessed with the Penn State Worry Questionnaire (Behar, Alcaine, Zuellig, & Borkovec, 2003; cut‐off for worry, Penn State Worry Questionnaire ≥ 45), panic symptoms with the Panic Disorder Severity Scale – Self‐Report (Houck, Spiegel, Shear, & Rucci, 2002; cut‐off for panic symptoms, Panic Disorder Severity Scale – Self‐Report ≥ 6), social anxiety symptoms with the Liebowitz Social Anxiety Scale – Self‐Rated (Baker, Heinrichs, Kim, & Hofmann, 2002; cut‐off for social anxiety, Liebowitz Social Anxiety Scale – Self‐Rated ≥ 30), symptoms of stress with the Perceived Stress Scale – 10 item (Cohen & Janicki‐Deverts, 2012; cut‐off for stress, Perceived Stress Scale – 10 item ≥ 13), insomnia symptoms with the Insomnia Severity Index (Bastien, Vallières, & Morin, 2001; cut‐off for insomnia, Insomnia Severity Index ≥ 8), and pain symptoms with the Multidimensional Pain Inventory (Kerns, Turk, & Rudy, 1985; cut‐off for pain, Multidimensional Pain Inventory ≥ 1.7).

### 2.3 Treatment components of TAIL

The treatment modules, or chapters, were distributed weekly or according to the participant's progress. The weekly treatment modules were mostly text based and included homework assignments with interactive work sheets. Each module ended with a homework report that the participant would complete and send to the therapist at the end of the week or when they were done. After feedback on the homework from the therapist, the participant would get access to the next module. The first three modules were mandatory and had a focus on depression and anxiety. These mandatory modules included identification of personal values, activity scheduling, and challenging of avoidance behaviours as main components. In the course of the first 3 weeks, the therapist phoned the participant once in order to discuss the selection of treatment modules for the tailored part of the treatment. Starting Week 4, the treatment content was tailored to the individual participant. One content option was to get more treatment components aimed at depression, such as more activity scheduling and the challenging of negative thoughts. Other content options were aimed at alleviating the symptoms of co‐occurring conditions. This included exposure with rationales for worry, panic, or social anxiety and interventions for stress, insomnia, or pain. The last module summarized the treatment and included relapse prevention planning. See Table 1 for a complete overview of the treatment modules available in TAIL.

### 2.4 Therapist contact

The therapists were licenced clinical psychologists or final‐year clinical psychology master students supervised by a licenced psychologist. The psychology students previously had 18 months of supervised face‐to‐face treatment with patients during their psychology training. Therapist support was mostly given as written messages in the treatment platform, and the therapists aimed at restricting support to 15 min per participant and treatment week. Written feedback was given after the completion of the homework assignments of every module. Participants also had the option to send messages to their therapist and receive an answer within 2 days. If a patient had been inactive for a week, they were prompted via a text message or a short phone call by the therapist to log in. In case of suicidal ideation, the therapists contacted the participant by phone and did an interview following the routines of the study.

### 2.5 Compliance scoring

The rating of compliance was done by two psychology master students who followed a structured guide for scoring, which was constructed for this study. Please see the Guide to compliance scoring in the Supporting Information. Inter‐rater reliability with intraclass correlation (two‐way mixed effects with absolute agreement) was first measured after 15 simultaneous ratings per module by the raters. Initially, there was an unsatisfactory agreement between raters for some modules, and the guide was then changed, or examples and comments were added to it. The second intraclass correlation analysis showed a satisfactory inter‐rater reliability of all the ratings of the treatment modules. Each homework component in the modules could generate a maximum score of 5 for a participant, meaning that the participant complied fully to that homework component, both quantitatively and qualitatively.

When the compliance with all homework components in all modules had been coded according to the reference guide, scores for Overall Compliance were constructed by summing up the compliance scores of all homework components for that participant. Then Specific Compliance scores for all six conditions were constructed by adding the compliance scores of the homework components in the modules for that condition, described in Table 1. Finally, there was a need to adjust for compliance to homework components that were not regarded as specific for any of the six conditions. Scores for Non‐Specific Compliance were therefore constructed for each condition by subtracting the Specific Compliance score for that condition, from the Overall Compliance score: Non‐Specific Compliance = Overall Compliance − Specific Compliance. Non‐Specific Compliance scores were later used to adjust for generic effects of compliance with treatment components not aimed at the analysed condition.

### 2.6 Statistical analyses

All statistical analyses were conducted in SPSS version 23 (IBM Corp.). Correlational analyses were used to explore if Overall and Specific Compliance predicted reductions on the symptom scales. Two hierarchical stepwise regression models were used for the whole group, with post‐treatment MADRS‐S as the dependent variable, and for each subgroup, with the condition‐specific symptom scale at post‐treatment as the dependent variable. In the first model, we explored Overall Compliance. Step 1 included the pretreatment symptom measure as a control, and in Step 2, the Overall Compliance measure was added. Step 3 constituted a sensitivity analysis that adjusted for age and sex. In the second model, compliance with specific homework components was explored in four steps. Step 1 was again to control for symptom score at pretreatment, and Step 2 added Specific Compliance as a predictor. Step 3 adjusted for Non‐Specific Compliance including compliance to generic and condition‐unrelated homework components. Step 4 was a sensitivity analysis that adjusted for age and sex. In order to investigate whether the specific homework components, aimed at the specific co‐occurring conditions, had an impact on the primary measure depressive symptoms, we also used the same second model in each subgroup again but with MADRS‐S as the dependent variable.

## 3 RESULTS

Descriptive statistics of the whole group and the six subgroups can be found in Table 2. Mean age (standard deviation; range) in the total group was 44.2 years (12.1; 19–67), and 77% were female. Participants with social anxiety had the highest Overall Compliance scores, and participants with pain had the lowest. All subgroups showed significant moderate to large reductions in the condition‐specific symptoms, except the pain subgroup where the effect on pain severity was small but still significant. For the whole sample and for the different subgroups, pretreatment symptom levels were tested between participants with and without missing postdata, and there were no major differences on symptom severity (please see Table S1).

**Table 2:** Subgroups with compliance scores, symptom scores, and effects on specific symptom scales

Subgroup	n	Overall Compliance, *M* [CI]	Specific Compliance, *M* [CI] (%)	Scale	Pre, *M* (*SD*)	Post, *M* (*SD*)	Pre–post test	Effect size (*g*)
Whole sample	207	34.7 [32.0, 37.3]	—	MADRS‐S	21.4 (7.0)	12.2 (8.8)	*t* = 15.59 *p* < 0.001	1.15
Worry	106	34.9 [31.2, 38.6]	6.2 [4.7, 7.7] (25% of max)	PSWQ	63.6 (9.1)	55.9 (10.9)	*t* = 7.92 *p* < 0.001	0.76
Panic	39	35.2 [28.3, 42.2]	5.1 [3.1, 7.1] (34% of max)	PDSS‐SR	11.5 (4.3)	6.7 (5.2)	*t* = 6.26 *p* < 0.001	1.01
Social anxiety	34	36.7 [29.1, 44.4]	3.0 [1.6, 4.5] (30% of max)	LSAS‐SR	71.9 (24.8)	54.1 (23.7)	*t* = 4.34 *p* < 0.001	0.73
Stress	131	35.0 [31.6, 38.3]	8.8 [7.3, 10.2] (25% of max)	PSS‐10	25.5 (5.3)	19.4 (7.2)	*t* = 9.15 *p* < 0.001	0.97
Insomnia	97	33.8 [29.9, 37.8]	3.2 [2.4, 4.0] (32% of max)	ISI	17.9 (4.4)	12.6 (6.6)	*t* = 7.77 *p* < 0.001	0.95
Pain	39	30.9 [24.6, 37.1]	3.8 [2.2, 5.4] (25% of max)	MPI	7.1 (2.9)	5.8 (3.7)	*t* = 3.67 *p* = 0.001	0.37

*Note*. MADRS‐S: Montgomery–Åsberg Depression Rating Scale – Self‐rated; PSWQ: Penn State Worry Questionnaire; PDSS‐SR: Panic Disorder Severity Scale – Self‐Report; LSAS‐SR: Liebowitz Social Anxiety Scale – Self‐Rated; PSS‐10: Perceived Stress Scale – 10 item; ISI: Insomnia Severity Index; MPI: Multidimensional Pain Inventory; *SD*: standard deviation; CI: 95% confidence interval; *g*: Hedge's *g*.

### 3.1 Analysis of Overall Compliance

The Overall Compliance scores predicted reductions in depression and explained 15% of the outcome variance (Table 3). In the subgroups of participants with specific conditions, Overall Compliance predicted the outcome on the worry, panic, social anxiety, stress, and insomnia symptom scales but not on the pain symptom scale. For participants with pain, pain symptoms at post‐treatment were only predicted by pain symptoms pretreatment. The sensitivity analysis, adjusting for age and sex in Step 3 (data not shown), did not change the results.

**Table 3:** Hierarchical regression analysis of Overall Compliance

Subgroup	Total group		Worry		Panic		Social anxiety		Stress		Insomnia		Pain	
Scale	MADRS‐S		PSWQ		PDSS‐SR		LSAS‐SR		PSS‐10		ISI		MPI	
	Δ*R* ^2^	β	Δ*R* ^2^	β	Δ*R* ^2^	β	Δ*R* ^2^	β	Δ*R* ^2^	β	Δ*R* ^2^	β	Δ*R* ^2^	β
Step 1	0.20***		0.27***		0.24**		0.26**		0.08**		0.09**		0.68***	
Pre score		0.45***		0.52***		0.49**		0.51**		0.29**		0.29**		0.82***
Step 2	0.15***		0.03*		0.18**		0.20**		0.19***		0.21***		0.00	
Pre score		0.41***		0.55***		0.43**		0.40**		0.32***		0.27**		0.81***
Overall Compliance		−0.39***		−0.17*		−0.43**		−0.46**		−0.43***		−0.46***		−0.03

*Note*. Outcome is post‐treatment score. Step 1 adjusts for pretreatment score. Step 2 adds Overall Compliance as a predictor. Step 3 (sensitivity analysis) not shown. MADRS‐S: Montgomery–Åsberg Depression Rating Scale – Self‐rated; PSWQ: Penn State Worry Questionnaire; PDSS‐SR: Panic Disorder Severity Scale – Self‐Report; LSAS‐SR: Liebowitz Social Anxiety Scale – Self‐Rated; PSS‐10: Perceived Stress Scale – 10 item; ISI: Insomnia Severity Index; MPI: Multidimensional Pain Inventory.

* *p* < 0.05.

** *p* < 0.01.

*** *p* < 0.001.

### 3.2 Analysis of Specific Compliance

The Specific Compliance scores predicted reductions in the corresponding specific symptoms for participants with social anxiety, stress, and insomnia but not for worry, panic, and pain (Table 4). Specific Compliance for social anxiety, stress, and insomnia still predicted symptom reductions when we adjusted for Non‐Specific Compliance in Step 3. Non‐Specific Compliance predicted symptom reductions in worry, panic, stress, and insomnia. In the sensitivity analysis (Step 4, data not shown), sex appeared as a significant predictor of symptom reductions in insomnia (males did better, *p* = 0.025). However, adjusting for Non‐Specific Compliance and demographic variables did not change the results that Specific Compliance was a strong and significant predictor of outcome for social anxiety, stress, and insomnia. When using the same model but with the depression scale as the dependent variable (Table 5), we found that Specific Compliance with the stress and insomnia components was significant predictors of reductions in depression symptoms. These analyses remained significant when we adjusted for Non‐Specific Compliance, age, and sex (Step 4, data not shown).

**Table 4:** Hierarchical regression analysis of Specific Compliance in the subgroups with specific conditions

Subgroup	Worry		Panic		Social anxiety		Stress		Insomnia		Pain	
Scale	PSWQ		PDSS‐SR		LSAS‐SR		PSS‐10		ISI		MPI	
	Δ*R* ^2^	β	Δ*R* ^2^	β	Δ*R* ^2^	β	Δ*R* ^2^	β	Δ*R* ^2^	β	Δ*R* ^2^	β
Step 1	0.27***		0.24**		0.26**		0.08**		0.09**		0.68***	
Pre score		0.52***		0.49**		0.51**		0.29**		0.29**		0.82***
Step 2	0.00		0.01		0.22**		0.17***		0.16***		0.00	
Pre score		0.53***		0.49**		0.47**		0.33***		0.42***		0.82***
Specific Compliance		−0.04		−0.07		−0.48**		−0.42***		−0.42***		0.02
Step 3	0.03*		0.19**		0.06		0.03*		0.09**		0.00	
Pre score		0.53***		0.41**		0.41**		0.33***		0.35***		0.76***
Specific Compliance		0.01		0.01		−0.39**		−0.33***		−0.31**		0.06
Non‐Specific Compliance		−0.19*		−0.45**		−0.26		−0.20*		−0.32**		−0.08

*Note*. Outcome is post‐treatment score. Step 1 adjusts for pretreatment score. Step 2 adds Specific Compliance as a predictor. Step 3 adjusts for Non‐Specific Compliance. Step 4 (sensitivity analysis) not shown. PSWQ: Penn State Worry Questionnaire; PDSS‐SR: Panic Disorder Severity Scale – Self‐Report; LSAS‐SR: Liebowitz Social Anxiety Scale – Self‐Rated; PSS‐10: Perceived Stress Scale – 10 item; ISI: Insomnia Severity Index; MPI: Multidimensional Pain Inventory.

* *p* < 0.05.

** *p* < 0.01.

*** *p* < 0.001.

**Table 5:** Hierarchical regression analysis of Specific Compliance with specific components in the subgroups with specific conditions

Subgroup	Worry		Panic		Social anxiety		Stress		Insomnia		Pain	
Scale	MADRS‐S		MADRS‐S		MADRS‐S		MADRS‐S		MADRS‐S		MADRS‐S	
	Δ*R* ^2^	β	Δ*R* ^2^	β	Δ*R* ^2^	β	Δ*R* ^2^	β	Δ*R* ^2^	β	Δ*R* ^2^	β
Step 1	0.19***		0.35***		0.37***		0.14***		0.25***		0.19**	
Pre score		0.44***		0.59***		0.61***		0.37***		0.50***		0.43**
Step 2	0.02		0.01		0.06		0.15***		0.07**		0.00	
Pre score		0.44***		0.56***		0.56***		0.29***		0.49***		0.43**
Specific Compliance		−0.14		−0.11		−0.24		−0.40***		−0.26**		0.03
Step 3	0.13***		0.14**		0.06		0.04*		0.10***		0.17**	
Pre score		0.39***		0.51***		0.59***		0.30***		0.42***		0.32*
Specific Compliance		−0.04		−0.07		−0.15		−0.30**		−0.18*		0.15
Non‐Specific Compliance		−0.38***		−0.38**		−0.25		−0.21*		−0.34***		−0.44**

*Note*. Outcome is post‐treatment score on MADRS‐S. Step 1 adjusts for pretreatment score. Step 2 adds Specific Compliance as a predictor. Step 3 adjusts for Non‐Specific Compliance. Step 4 (sensitivity analysis) not shown. MADRS‐S: Montgomery–Åsberg Depression Rating Scale – Self‐rated.

* *p* < 0.05.

** *p* < 0.01.

*** *p* < 0.001.

## 4 DISCUSSION

The current study examined overall and condition‐specific homework compliance as predictors of reduction in a wide range of symptoms in patients with depression and co‐occurring conditions, undergoing an individually tailored internet‐based treatment. Overall Compliance with the homework assignments was associated with a reduction in symptoms of depression (explained variance 15%) but also in symptoms of worry (3%), panic (18%), social anxiety (20%), stress (19%), and insomnia (21%), for participants with these co‐occurring conditions. The association of Overall Compliance and the reduction in pain symptoms were not significant. These associations are, except for worry and pain, large in comparison with results from homework compliance studies in face‐to‐face treatment where a meta‐analysis reported an average association of 7% explained variance (Mausbach et al., 2010). In a larger context, our results strengthen the picture of compliance to homework assignments as one of the most important aspects in internet‐based treatment, for example, when comparing the results with the estimated association of 8% between working alliance and outcome in internet‐based treatment, which was presented in a recent meta‐analysis (Flückiger, Del Re, Wampold, & Horvath, 2018).

For participants with social anxiety, stress, and insomnia, compliance with the specific homework components targeting those conditions was also associated with reductions in the corresponding symptoms. On the other hand, Specific Compliance did not predict specific reductions for participants with worry and panic. For participants with pain, neither Overall nor Specific Compliance predicted reductions in pain symptoms. Compliance with specific homework components targeting stress and insomnia also predicted reductions on depression, for participants with these conditions.

### 4.1 Interpretations

These exploratory findings are accompanied by some interpretations for each condition below.

#### 4.1.1 Worry

For participants with worry, pretreatment score was the strongest predictor of outcome, but there was also a small effect of Overall Compliance with the treatment material. Because Specific Compliance with the components targeting worry did not seem to have an effect, this result suggests that a transdiagnostic approach, including homework components such as activity scheduling and challenging avoidance behaviours, could be enough for participants with worry.

#### 4.1.2 Panic

For participants with panic disorder symptoms, the results indicated a relatively large dose–response effect of complying to the homework components, but this did not have to be to the panic disorder‐specific components. Earlier research has suggested that good‐quality exposure is a strong predictor of outcome in psychological treatment for panic disorder (Cammin‐Nowak et al., 2013). Because some material on avoidance behaviour and exposure therapy was included in the three initial mandatory modules of the treatment, this could have had a specific effect on panic symptoms but would still be counted as Non‐Specific Compliance for panic disorder in our statistical models. However, because the subgroup of participants with panic disorder symptoms was small, these results have to be interpreted with caution.

#### 4.1.3 Social anxiety

For social anxiety, as for panic, the results suggested a possibly large dose–response relationship of compliance and outcome, but for social anxiety, it was complying to the specific social anxiety homework components that predicted a better outcome. This suggests that individuals with social anxiety may benefit from working with components that are clearly aimed at social anxiety, such as shifting focus outwards and exposure to social situations. However, because the subgroup of participants with social anxiety in this study was small, these results also have to be interpreted with caution.

#### 4.1.4 Stress

Participants with high levels of stress seemed to benefit from Overall Compliance, but the largest predictive effects on outcome were from compliance with specific stress homework components, for example, planned recovery and exposure to not doing things perfectly. Compliance with the stress components was also important for the reduction of depression symptoms for these patients. Stressed participants in primary care may often receive treatment for depression and may thus not benefit from, or recognize themselves in, a treatment lacking content specifically about stress. A high percentage of participants in the study suffered from depression with high amounts of stress, and it seems that they benefited extra from content tailored to help them handle their stress‐related problems.

#### 4.1.5 Insomnia

Also for participants with insomnia, the results indicated a large dose–response relationship of Overall Compliance and symptom reduction. When separating Specific from Non‐Specific Compliance, both seemed to predict outcome. The finding that Specific Compliance was a significant predictor strengthens earlier research suggesting that specific insomnia treatment components, such as sleep restriction and stimulus control, are important in treatment of insomnia (Kaldo et al., 2015). Compliance with the specific insomnia components also predicted the reduction of depression symptoms for participants with insomnia, suggesting the importance of alleviating insomnia symptoms to improve depression symptoms, which has been seen in previous research on internet‐based treatment (Blom et al., 2015).

#### 4.1.6 Pain

The participants with pain did not improve much on pain symptoms, but they did experience reductions in other symptoms, such as depression. Participants with higher initial levels of pain did not reduce their pain regardless of complying to the pain treatment modules. This suggests that the pain components in the current treatment were without effects on pain and that these participants may instead need a dedicated pain treatment, which have shown small to moderate effects in previous studies (Buhrman, Gordh, & Andersson, 2016).

### 4.2 Limitations and strengths

This study has some strengths and limitations. Limitations include that we performed explorative analyses based on correlational data. The absence of an experimental control makes these findings preliminary. The possible existence of confounding variables, influencing both compliance and outcome, cannot be completely controlled for in a correlational design. It is partly managed by controlling for Non‐Specific Compliance when analysing Specific Compliance and by some sensitivity analyses, but this still cannot compensate for the lack of an experimental design. There is however previous research on face‐to‐face psychological treatments suggesting that homework compliance causes reductions in depression rather than the other way around (Burns & Spangler, 2000), although this research cannot rule out other causal mechanisms.

The number of participants with panic disorder symptoms and social anxiety was small, resulting in low statistical power for these conditions. However, the strong statistical effect of compliance to specific social anxiety components seems to be an indicator of a substantial association in that case.

Another limitation, seen in the analysis of compliance with the panic disorder components, is that the components in the introductory modules contained homework assignments, for example, exposure therapy and challenging negative thoughts, that could also have a condition‐specific effect and thus leave less room for further improvement for the following disorder‐specific modules. This sometimes unclear separation of components calls for caution for some interpretations. For example, the result that specific modules for panic disorder did not further improve panic disorder cannot be interpreted as evidence that panic‐specific components are not useful.

The strengths of this study include that we created and employed a measure of treatment compliance where both quantity and quality of compliance were systematically rated, as recommended by Kazantzis et al. (2017), and most likely providing a better estimate of treatment compliance than more basic measures, such as number of accessed modules. Other strengths are a large total sample, which is representative of depression patients in primary care.

### 4.3 Future directions

These findings support the idea that qualitative and quantitative measures of compliance with internet‐based treatment are of value, especially for individually tailored treatments, when one wishes to assess the maximum efficacy of different treatment components. Future studies could examine compliance with specific treatment components in a randomized setting to investigate the direction of causality. Future studies could also examine how other concepts, such as treatment credibility, or how much time and effort the participants put into the treatment, relate to treatment compliance.

## 5 CONCLUSIONS

Overall homework compliance was important for outcome in this individually tailored internet‐based treatment for depression in a sample with high levels of co‐occurring conditions. Compliance with specific homework components for social anxiety, stress, and insomnia was important to alleviate the symptoms of these specific conditions. Compliance with homework components for stress and insomnia was important also for reducing depression levels, for participants who presented with these conditions. Because a high degree of participants in the study suffered from stress and insomnia, and these participants benefited extra from complying to the stress‐specific and insomnia‐specific components, the inclusion of tailored content for these conditions should probably be a priority when choosing components to include in interventions for patients with high levels of co‐morbid symptoms.

## CONFLICT OF INTEREST

We wish to confirm that there are no known conflicts of interest associated with this publication and there has been no significant financial support for this work that could have influenced its outcome.

## Supporting information


**Table S1.** Missing data analysis.

Guide to compliance scoring
