# Supplementary material for: The effects of component‐specific treatment compliance in individually tailored internet‐based treatment
Source: Clin Psychol Psychother. 2019 Feb 22;26(3):298–308. doi: 10.1002/cpp.2351 (PMC6635903; doi:10.1002/cpp.2351)
Supplement: Supplementary file 1 — Table S1. Missing data analysis. [file CPP-26-298-s001.docx]

**Supplementary table 1.** Missing data analysis.

Total group and subgroups with comparison of demographics, compliance scores and pre-treatment scores between participants included and excluded from regression analysis because of missing values at post treatment.

| Sub-group |  | *n* | Age m(SD) | Proportion female | Overall Compliance m[CI] | Specific Compliance m[CI] | Scale | Pre score m(SD)[CI] |
| --- | --- | --- | --- | --- | --- | --- | --- | --- |
| Total group | Complete | 207 | 44.2 (12.1) | 159 (77%) † | 34.7 [32.0-37.3] * | - | MADRS-S | 21.4 (7.0) [20.4-22.4] |
|  | Missing | 85 | 41.1 (12.8) | 51 (60%) | 6.75 [4.7-8.8] |  |  | 22.7 (6.9) [21.0-24.4] |
| Worry | Complete | 106 | 43.0 (11.9) | 81 (76%) | 34.9 [31.2-38.6] * | 6.2 [4.7-7.7] * | PSWQ | 63.6 (9.1) [61.8-65.3] |
|  | Missing | 39 | 40.6 (12.6) | 23 (59%) | 8.2 [4.8-11.6] | 1.0 [-0.1-2.1] |  | 64.9 (9.2) [61.9-67.9] |
| Panic | Complete | 39 | 43.0 (11.7) | 26 (67%) | 35.2 [28.3-42.2] * | 5.1 [3.1-7.1] * | PDSS-SR | 11.5 (4.3) [10.2-12.9] |
|  | Missing | 15 | 37.7 (13.4) | 11 (73%) | 10.1 [3.6-16.5] | 1.9 [-0.3-4.2] |  | 13.3 (4.8) [10.6-15.9] |
| Social anxiety | Complete | 34 | 45.0 (12.6) * | 26 (76%) † | 36.7 [29.1-44.4] * | 3.0 [1.6-4.5] | LSAS-SR | 71.9 (24.8) [63.3-80.6] |
|  | Missing | 12 | 31.7 (11.4) | 5 (42%) | 10.2 [2.2-18.2] | 1.3 [-0.5-3.0] |  | 66.5 (18.6) [54.7-78.3] |
| Stress | Complete | 131 | 43.9 (12.1) * | 105 (80%) † | 35.0 [31.6-38.3] * | 8.8 [7.3-10.2] * | PSS-10 | 25.5 (5.3) [24.6-26.4] |
|  | Missing | 47 | 39.5 (12.2) | 29 (62%) | 7.4 [4.6-10.2] | 0.4 [0.1-0.7] |  | 27.0 (5.5) [25.4-28.6] |
| Insomnia | Complete | 97 | 44.4 (11.8) | 74 (76%) | 33.8 [29.9-37.8] * | 3.2 [2.4-4.0] * | ISI | 17.9 (4.4) [17.0-18.8] |
|  | Missing | 30 | 42.2 (13.6) | 18 (60%) | 6.9 [3.3-10.5] | 0.3 [0.0-0.6] |  | 19.2 (5.0) [17.4-21.1] |
| Pain | Complete | 39 | 46.9 (10.1) | 31 (79%) | 30.9 [24.6-37.1] * | 3.8 [2.2-5.4] | MPI | 7.1 (2.9) [6.1-8.0] |
|  | Missing | 10 | 44.6 (17.4) | 6 (60%) | 7.5 (1.6-13.5] | 0.7 [-0.4-1.8] |  | 8.0 (1.7) [6.8-9.2] |

PSWQ, Penn State Worry Questionnaire; PDSS-SR, Panic Disorder Severity Scale – Self report; LSAS-SR, Liebowitz Social Anxiety Scale – Self-Rated; PSS-10, Perceived Stress Scale – 10 item; ISI, Insomnia Severity Index; MPI, Multidimensional Pain Inventory; SD, standard deviation; CI, 95% confidence interval; * = T-test significant *p* < .05; † = Chi 2-test significant *p* < .05.
