# Supplementary material for: The effects of component‐specific treatment compliance in individually tailored internet‐based treatment
Source: Clin Psychol Psychother. 2019 Feb 22;26(3):298–308. doi: 10.1002/cpp.2351 (PMC6635903; doi:10.1002/cpp.2351)
Supplement: Supplementary file 2 — Guide to compliance scoring [file CPP-26-298-s002.docx]

**Guide to compliance scoring**

The maximum compliance score per treatment module is 5. For every module the following scoring procedure was followed:

**a)** **Received module.** Score 1 if the participant has received the module.

**b)** **Quantity.** For the modules that the participant has received, score:

0: for not handing anything in from the module.

1: for handing in something, but not enough (for example answered the knowledge quiz but has not done the homework task, or has done the homework task but not answered the knowledge quiz).

2: for handing in and done enough. Enough means that the participant should have answered the knowledge quiz and in most of the cases done a substantial part (approximately >80 %) of the homework task and described this in a work sheet.

**c)** **Quality.** For all the modules that the participant has received, also score:

-1 = if there are clear indications that the participant has done the module but experienced failure, received a negative effect or is dissatisfied.

0 = if the work was not done, or neither the participant nor the therapist expressed any opinion about how well the work went.

1 = if the participant and the therapist thought the work went well to some extent, or that the therapist thought it went well, but the participant expressed doubts.

2 = if the participant reported that the work with the module went fine, and the therapist expressed that he/she was pleased with the work of the participant.
